# Green Smart Campus Monitoring and Detection Using LoRa

**DOI:** 10.3390/s21196582

**Published:** 2021-10-01

**Authors:** Kuo-Hsiung Tseng, Meng-Yun Chung, Li-Hsien Chen, Pei-Yao Chang

**Affiliations:** 1 Department of Electrical Engineering, National Taipei University of Technology, Taipei 10608, Taiwan; f10473@mail.ntut.edu.tw (K.-H.T.); alexmychung@gmail.com (M.-Y.C.); 2 Department of Civil Engineering, National Taipei University of Technology, Taipei 10608, Taiwan; 3 Industrial IoT, Advantech, Taipei 10608, Taiwan; jim98765590@gmail.com

**Keywords:** LoRa, wireless communication, sensor, Internet of Things, monitoring system

## Abstract

Along with the rapid development of sensing systems and wireless transmission technology, the scope of application of the IoT has substantially increased, and research and innovation that integrate artificial intelligence. This study integrated civil engineering and electrical engineering to establish a universal and modularized long-term sensing system. Aiming at positive construction in civil engineering, the campus of National Taipei University of Technology was used as the experimental site as a green campus. This paper focused on the cooling effect of the green roof and the temperature difference of the solar panel to effectively isolate the direct sunlight on the roof of the building. To achieve long-term monitoring, energy consumption must be minimized. Considering that the distance between sensor nodes in the experimental site was over dozens of feet, LoRa transmission technology was selected for data transmission. LoRa only consumes a small amount of energy during data transmission, and it can freely switch between work modes, achieving optimal power utilization efficiency. The greening-related research results indicated that the shade from solar panels on the rooftop could effectively reduce the temperature increase caused by direct sunlight on concrete surfaces. The temperature reduction effect was positively correlated with whether the solar panels provided shade. After 1 week of monitoring, we observed that having plants on the rooftop for greening negatively correlated with temperature reduction efficiency. Permeable pavement on the ground was positively correlated with temperature reduction efficiency. However, its temperature reduction efficiency was inferior to that of solar panel shading. The temperature difference between high-rise buildings and the ground was approximately 1–2 °C. At the same elevation, the temperature difference between buildings with and without greening was approximately 0.8 °C. Regarding the sensing system designed for this site, both hardware and software could be flexibly set according to the research purposes, precision requirements of the sites, and the measurement scope, thereby enabling their application in more fields.

## 1. Introduction

In recent years, people have begun to reflect on previously overlooked problems, such as the greenhouse effect, damage to the environment and ecology, personal safety, and health care [1]. An increasing number of research teams are using long-term monitoring data for research and analysis to predict various challenges that nature may present in the future [2,3,4]. For example, gas exhaust from producing technology products increases the greenhouse gas content in the atmosphere [5,6]. Infrared radiation absorbs and retains the energy in the exhaust from burning fossil fuels and in water vapor, carbon dioxide, and methane. This causes the surface temperature worldwide to increase, exacerbating the greenhouse effect, causing global warming, and affecting the ecology and environment. To combat these effects, the concept of sponge cities is being applied. Sponge cities refers to cities with favorable flexibility when encountering severe natural disasters [7,8,9]. That is, they have the capacity to absorb water, retain water, clean water, filter air, and filter pollutants; the beneficial effects of these features include temperature reduction, flood prevention, drought prevention, and carbon storage. These capabilities can help address the problem of cities damaging water supplies and ecology, resulting in cities that coexist with the environment and have a low carbon footprint. However, the establishment of a sponge city is based on several key concepts. Moreover, quantitative data are required to verify the effectiveness of the greening engineering in a sponge city. Therefore, sensing elements must be added to all engineering methods to record real-time information [10,11]; moreover, cloud servers can be employed for analysis and to provide greening results with more favorable readability and reliability. Some methods to make cities greener include the following:

1. City surfaces can be altered to include permeable pavement that can breathe. Like the capillary pores of the human body, such pavements allow water to penetrate. In this manner, life can flourish under the surface. When a concrete city no longer blocks the Earth from being in contact with nature, humans and the environment can coexist and help each other, and disasters can be minimized.
2. Greening the environment to reduce carbon dioxide concentration is another method to prevent the exacerbation of the greenhouse effect. Abundant vegetation planted on city rooftops can consume carbon dioxide, reducing the burden of a concrete city on the environment as well as the impact of the heat island effect.
3. Using natural forms of energy, such as solar power and wind power, to replace energy generated through burning coal or oil can also reduce the generation of greenhouse gas.

In the early stages of the Internet, network communication relied heavily on cables, and the transmission distance was restricted by cable length. However, the wireless local area network was developed in 1997. In that year, the IEEE (International Institute of Electrical and Electronic Engineering) formulated the 802.11 wireless network specification. It began to not use conductors or cables to set up wireless network domains. Instead, it used similar radio transmission and modulation signal processing methods to form strong penetrating power and all-round transmission [12]. Following the introduction of wireless internet, conductors or cables were no longer necessary to establish wireless domains. In subsequent years, wireless transmission technology bloomed. Transmission protocols such as Bluetooth, Wi-Fi, and Zigbee have been developed, and the frequency has been increased from the original 2.4 GHz to 5 GHz [13,14]. The transmission distance has also gradually increased, and the transmission speed has increased 10-fold because of modulation technology—from 2 Mbps to 1.3 Gbps. LoRa has become one of the most effective solutions in the field of the Internet of Things (IoT) as serving some of the most significant features, such as low cost and low power wireless platforms. LoRa technology uses the LoRa Wide Area Network (LoRaWAN) protocol to solve several types of real-life problems [15]. As the Internet of Things (IoT) is rapidly adopted worldwide, the demand for wireless communication technology has increased tremendously, leading to the increased focus on research and development of wireless transmission technology. Research teams have aimed to develop technology that exhibits low power consumption, long-distance transmission capability, and high transmission speeds for various purposes. For example, environmental sensing requires low power consumption and long-distance transmission because researchers cannot frequently enter the research site. Smart homes require high transmission speed as well as expandability and flexibility. In terms of application scope and site, the IoT can be implemented at places such as homes and factories and even in the natural environment. In recent years, governments and many organizations around the world have worked hard to promote the importance of reducing energy consumption and carbon emissions, and have begun to advocate the concept of the “Green Internet of Things”, with applications in areas such as forestation zones, greenhouses, green energy management, farmland, and forest areas [16,17].

This study integrated civil engineering and electrical engineering to establish a universal and modularized long-term sensing system. This study adopted the notion of green smart campus to discuss the cooling effect of green rooftops and the temperature differences caused by the shade from solar panels on building rooftops. This study focused on system development and the construction of the sensing system end, including adjusting the antenna transmission power, sleep and standby state, preprocessing of multiple physical quantity data, and data return calibration.

## 2. Materials and Methods

### 2.1. Establishing Sensing Elements

The IoT architecture of this experiment is mainly divided into the perception layer, network layer, and application layer. In terms of the perception layer, this study uses a microcontroller to present the data of the temperature and humidity sensors. In the network layer, the packet is transmitted to the receiving module through LoRa transmission technology, and the receiving module is additionally loaded with an Ethernet interface to transmit the data to the local server using the ethernet network. In the application layer, the returned data is stored in the database, and finally the content need to be displayed is displayed on the monitoring webpage. Because the research site of this study was an actual campus, this study used temperature and humidity sensors that met relevant demands such as the temperature range, adaptability, and the moderate precision and resolution of the research subjects were considered. The research site was divided into three categories: above ground and underground of the permeable pavement, shade of the solar panels, and greening rooftop. The measurement parameters were the temperature and humidity felt by the human body. 

SHT10 is a patch package sensor type that consists of two parts: the sensing element and the signal processing circuit. The designs are located on a microcircuit board. The output is a designated digital signal. The sensor employs the patented CMOS technology to ensure that it has extremely high reliability and stability. The sensor consists of a capacitive polymer humidity measuring element and a temperature measuring element, a 14-bit analog to digital converter, and a serial interface. The sensor leverages the fact that dielectrics can absorb or release water molecules to maintain a positive correlation with the relative environmental humidity. The change in the capacitance capacity can be measured using an electronic circuit, in turn enabling the measurement of the relative humidity in the air. The CMOS technology involves a layer of protective polymer covering a finger electrode system, thereby enabling the capacitance function. The technology improves the characteristics of a sensor and shields the sensor from external influence. Before the temperature and humidity sensors were buried in the soil to measure soil temperature and humidity, they were covered in casings. The ideal temperature measurement range was between 0 °C and 40 °C, and the temperature range in which the humidity had the smallest bias was between 20 °C and 80 °C. Table 1 presents detailed information on the parameters. 

In the aforementioned description, relative humidity refers to the ratio of absolute humidity to the maximum humidity, representing the water vapor content in the environment. When relative humidity reaches 50%, the air contains half of the water vapor of the air saturation point (100% relative humidity) at the same temperature (25 °C). When relative humidity exceeds 100%, water vapor condenses. As the temperature increases, the amount of water the air can contain increases. Therefore, temperature changes alter relative humidity. Thus, to calculate humidity in a study, temperature must be included as a parameter. Using humidity and temperature, dew point temperature can be calculated.

(1) $$\rho_{w}=\frac{m}{v}$$

(2) $$\%\mathrm{RH}=\frac{\rho_{w}}{\rho_{w,max}}$$

(3) $$\rho_{w,max}\propto T$$

where

$\rho_{w}$ is absolute humidity, or the amount of water vapor contained in a unit volume,

m is the amount of water vapor,

v is volume,

% RH is relative humidity, or the ratio of absolute humidity to the maximum humidity, and

$\rho_{w,max}$ is the maximum humidity, which is proportional to the air temperature.

Dew point temperature refers to the temperature required for water in the gaseous state in the air to reach saturation and condense into liquid state water under a fixed barometric pressure [18]. At this temperature, the condensed water floating in the air is called fog, and its attachment to the surface of a solid object is called dew, resulting in the name “dew point temperature.” By measuring humidity and temperature, a precise measurement of the dew point can be obtained. In this study, we adopted the Pearson product-moment correlation formula to measure the test-retest reliability of the sensor. The Pearson product-moment correlation formula is as follows:

(4) $$\gamma_{R}=\frac{\sum \left(x-\bar{x}\right)\left(y-\bar{y}\right)}{\sqrt{\sum {\left(x-\bar{x}\right)}^{2}}*\sqrt{\sum {\left(y-\bar{y}\right)}^{2}}}$$

where 

$\gamma_{R}$ = reliability indicator

x = measured temperature 

$\bar{x}$ = mean of the measured temperatures

y = test temperature

$\bar{y}$ = mean of the test temperatures 

**Figure 1:**
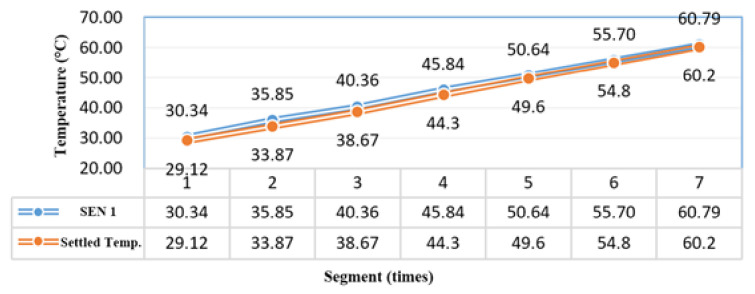
Temperature increase trend of Sensor 1.

Relevant statistics of the sensor are listed in Table 2, and the data are presented in a trend graph in Figure 1. The temperature measured by the sensor differed by approximately 1 °C from that measured by the electric thermometer. The test–retest reliability method was subsequently adopted. $\gamma_{R}=$ 99.95%, where ① = $\sum \left(x-\bar{x}\right)\left(y-\bar{y}\right)$, ② = $\sqrt{\sum {\left(x-\bar{x}\right)}^{2}}$, and ③ = $\sqrt{\sum {\left(y-\bar{y}\right)}^{2}}$. 

### 2.2. LoRa Communication Module Setting

The promotion of Industry 4.0 has led to exponential growth in the IoT. However, the IoT, which has been utilized by various fields, is mainly based on wireless transmission technology, and its increased application causes wireless transmission technology such as Bluetooth, Wi-Fi, Thread, Zigbee, and 4G to receive increasing attention from research teams and the public [19]. Each of these wireless transmission protocol has distinct advantages and disadvantages. Thus, the specific considerations of the implementation sites determine which technology is applied. For example, Bluetooth transmission uses digital modulation technology to increase its interference resistance. It also features coding encryption for security protection. Bluetooth data transmission speeds can reach 1 Mbit/s. Because of its limited transmission distance, typically less than 10 m, this technology is suitable for small areas such as smart homes. Zigbee can transfer data over distances up to 100 m. By increasing its transmission power, the distance can reach several hundred meters or even nearly 1 km. The data transmission speed of Zigbee is 250 kbit/s [20]. Currently, it has a large market share in wireless illumination. Zigbee is most effective for transmitting small amounts of data over short distances. Thread is a technology similar to Zigbee. It is also used in short-range transmission, such as in smart homes, and it can integrate multiple devices. Wi-Fi, used for the transmission of large amounts of data, is convenient for use in portable devices. Its disadvantage is that it consumes more energy than other wireless transmission technologies [21]. Moreover, use of a mobile network necessitates the use of a SIM card, which is not suitable for networks with many devices. Therefore, for situations where low power consumption and long-distance transmission are required, such as for long-term biotracking in an environment, LoRa technology was developed. The name LoRa is derived from the combination of “long range.” The major advantage of LoRa is long-distance transmission [22]. With a single signal receiving and transmission base station, an area of several kilometers is covered. In terms of technology, its wireless assembly is focused on satisfying long-distance communication. Numerous conventional wireless systems are modulated using physical layer frequency-shift keying because it is a high-efficiency and low power consumption approach. Bluetooth, another wireless transmission protocol, has a transmission distance of approximately 60 feet (or 20 m). By contrast, that of Wi-Fi is 200 feet (or 60 m). To increase the packet transmission range as well as transmission speed of these technologies, power consumption must be increased, consequently reducing battery life. Therefore, a key feature for products such as LoRa is low power consumption along with long-distance data transmission. 

This study selected the module designed using the SEMTECH SX1278 chip. Because the experimental site was in an urban campus full of buildings, the module with 1 watt transmission power was selected to ensure the effective return of sensing data. The working band was set between 410 and 441 MHz, which could be adjusted and assessed. The detailed specifications are presented in Table 3.

The LoRa transmission module selected in this study had four work modes, namely general mode, wakeup mode, power-saving mode, and sleep mode. M0 and M1 were used to determine work modes. 

General mode: When M0 = 0 and M1 = 0, the module receives serial data. The user can enter 512 bytes of data; however, the length of the wireless packet transmitted by the module is 58 bytes. When the data in the buffer register reaches 58 bytes, the module automatically transmits a packet. At this point, the register can continue adding to the data length from the previous data entry. When the packet to be transmitted is smaller than 58 bytes, the module automatically waits for an interval sufficient for entering 3 bytes. If no packet data are entered, then it determines that the data have ended, and it transmits all packets wirelessly. Packets transmitted under the general mode can only be received by the receiving module under the general mode or the wakeup mode. When receiving, the module’s wireless receiving function is open. 

Wakeup mode: When M0 = 0 and M1 = 1, the packet transmission condition initiated by the module and the AUX function are identical to that of the general mode. The only difference is that the module automatically adds a wakeup code in each packet. The length of the wakeup code is determined by the wakeup time set in the parameter in the module setting. The purpose of the wakeup code is to wake up the receiving module that is in power-saving mode. Therefore, when the module is working in wakeup mode, the data transmitted can be received by modules in the general, wakeup, and power-saving modes. 

Power-saving mode: When M0 = 0 and M1 = 1, the module is in power-saving mode; that is, it enters a sleep state (which differs from the sleep mode.) The serial sequence is closed and cannot receive serial sequence data from the microcontroller. The mode regularly monitors for the wakeup code. Once it receives a valid wakeup code, the module remains in a receiving state until all valid packets are received. In this situation, the AUX transmits in a low frequency. After a 5 ms delay, it opens the serial sequence wireless data and transmits through TXD. Subsequently, it uses AUX to transmit in a high frequency. Next, the receiving module enters the sleep–monitoring state (polling). According to the wakeup time set, the module exhibits a different receive response delay (up to 2 s) and mean receiving current (minimum of 30 uA; Table 4). 

**Figure 2:**
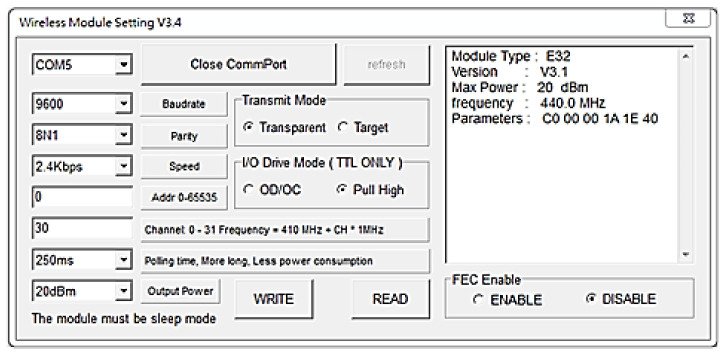
LoRa module parameter setting.

Sleep mode: When M0 = 1 and M1 = 1, sleep mode is active. During sleep mode, packets cannot be transmitted or received. This mode is for setting parameters. The module parameters are entered according to the series sequence (Figure 2). Module parameter is as shown in Table 5. Module parameters can be set directly or by using computer software. They are set according to the research purposes. When a short response time is required, then wakeup code parameters are set. When low power consumption is required, then transmission power parameters are set. When data packet accuracy is demanded, then the transmission rate is adjusted. 

### 2.3. Site Testing Regions

**Figure 3:**
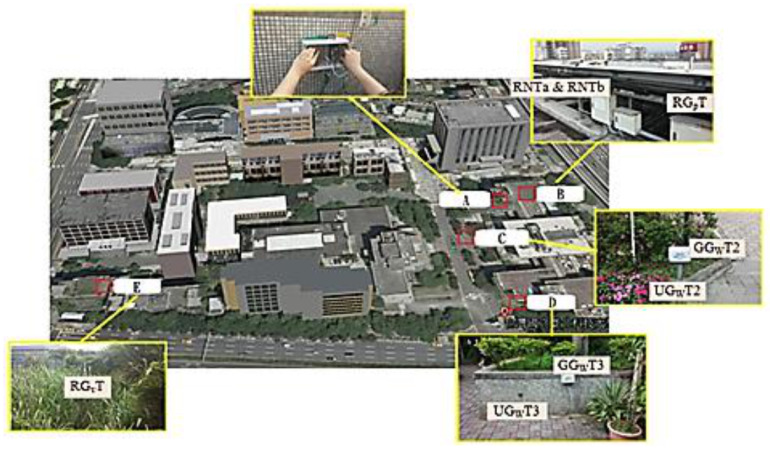
Sensor distribution (modified from Google Maps). The explanation for (**A**–**E**) is in Table 6.

As indicated in Figure 3, the equipment installation was divided into two parts. The first part involved the installation of the hot spots for data collection. The hot spots consisted of the outer box of the waterproof room, the physical network communication wires, data transmission and receiving modules, and power source allocation. In the construction principle, since the signal will weaken the energy due to crossing obstacles, it must be installed outdoors. In order to achieve good transmission efficiency and consider the possibility of future expansion of research projects and the possibility of expanding the deployment field, the base will be collected. The platform is erected on the outer wall of the top floor of the North Building of the Integrated Technology Complex. (Region A). The second part involved sensor terminal equipment, which consisted of an outdoor box, antenna, transmission module, and power supply equipment. The sensors were installed in Regions B, C, D, and E (Figure 3) in accordance with the research purposes. The codes of each sensor are listed in Table 6.

Note:

Region A: Data collection hot spot devices in the North Building of the Integrated Technology Complex. The height is about eight floors high (24 m). The latitude and longitude is 121.5358 and 25.0431, separately.
Region B: Shade of the solar panel on the North Building of the Integrated Technology Complex. The height is about eight floors high (24 m). The latitude and longitude is 121.5358 and 25.0431, separately.
Region C: Type A permeable brick pavement in front of the Integrated Technology Complex. The height is level ground. The latitude and longitude is 121.5355 and 25.0429, separately.
Region D: Type B permeable brick pavement in front of the Zhongxiao Gate. The height is level ground. The latitude and longitude is 121.5358 and 25.0422, separately.
Region E: Greening area on top of the Design Building. The height is about seven floors high (21 m). The latitude and longitude is 121.5332 and 25.0427, separately.

## 3. Results and Discussion

### 3.1. Analysis of Data from the Shade of the Solar Panels

**Figure 4:**
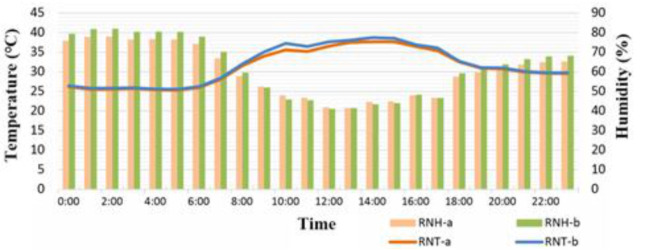
Comparison of RNH and RNT between a and b.

In Region B, this study installed two sets of sensors, namely RNT and RNH (each set containing two subsets, a and b, to verify their accuracy) and RGpT and RGpH (Figure 4). The purposes of these sensors were two-fold. First, they identified whether the data measured by these sensors were the actual temperature and humidity of the environment and ensured that the sensors remained in the working state. Second, having two sets minimized the possibility of missing sensing data because of sensor malfunctions. One set was used as backup. The Pearson correlation coefficient was used to calculate their similarity (S = 99.8%), and the mean difference was 0.3 °C. Data used in this study were obtained from the aforementioned sensing system. Data were collected and transmitted to the server for organization. Take the single-day data of 10 May of Region B in Table 7 as an example. The sampling duration of the data in the table was 1 h. The measuring time of each sensor in the system was 30 min. To avoid redundant data affecting the analysis results and discussion, the sampling duration was set at 1 h. Temperature was measured to two decimal places, and humidity was presented in percentage. Temperature values were presented in color gradient to illustrate changes. 

**Figure 5:**
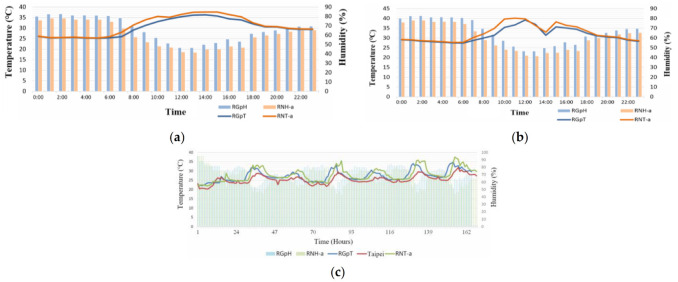
Temperature and humidity when solar panels are used to provide shade for temperature reduction. (**a**) 10 May, (**b**) 11 May, and (**c**) temperature reduction efficiency.

Figure 5a,b illustrate two sets of temperature curves, that of the area under the shade of the solar panel and that of the area under direct sun exposure on the rooftop, as well as their corresponding humidity bars. After 7:00 a.m., data from RGpT and RNT exhibited substantial differences. At 6:00 p.m., the two sets of data were similar. The temperature difference resulted mainly from whether the sun was shining directly on the sensor. Figure 5 presents the data of two consecutive days, 10 May and 11 May, exemplifying this phenomenon. A special situation is recorded in Figure 5: from 1:00 p.m. to 2:00 p.m. the temperature dropped substantially. According to the sunshine values measured by a solar photovoltaic power generation system established by this laboratory and the values measured by the Taipei Station of the Central Weather Bureau (CWB), from 1:00 p.m. to 2:00 p.m. on that day cloudy conditions prevailed, resulting in a sudden drop in the intensity of the sunshine and the corresponding drop in temperature. Figure 5c shows the temperature display in the one-week time interval of Region B. It can be seen whether the temperature and humidity in each area show the same temperature change as the temperature and humidity of the Taipei station of the Central Weather Bureau of Taiwan, and the degree of credibility and similarity can be judged. The encircled parts represent a duration of approximately 7–8 h. Comparison of the changes in the three curves indicated that the temperature changes were similar, with a temperature difference of approximately 2–3 °C. That is, the temperature and humidity data collected by this system is highly reliable. As for similarity analysis, it is known by using the cosine similarity theorem, Equation (5), where cosθ is represented by the code S (Similarity), and the respective similarity indexes will be obtained after pairwise comparison. Regarding the waveform differences presented by the curves of RGpT and RNT, the temperature was affected by whether the rooftop of the building was shaded. Shade could reduce the outdoor temperature and prevent the indoor temperature from increasing. Table 8 presents the calculation of the similarities of the temperatures and humidity in Region B.

(5) $$\cos\theta =\frac{\sum_{k=1}^{n}x_{1k\;}\cdot x_{2k\;}}{\sqrt{\sum_{k=1}^{n}x_{1k\;}{}^{2}}\cdot \sqrt{\sum_{k=1}^{n}x_{2k\;}{}^{2}}}$$

### 3.2. Analysis of Permeable Brick Pavement Data

**Figure 6:**
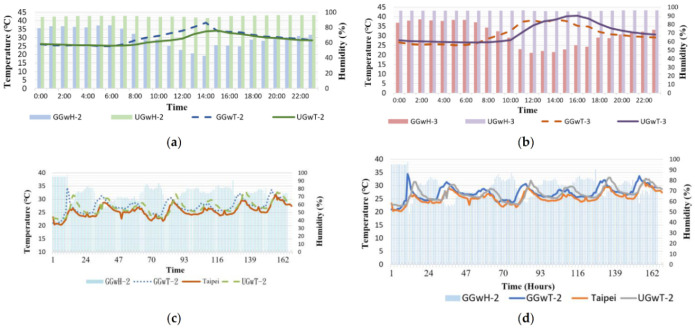
Temperature and humidity of permeable brick pavement (**a**) Region C, (**b**) Region D, (**c**) temperature reduction effect in Region C, and (**d**) temperature reduction effect in Region D.

In Regions C and D, this study installed sensors GG_W_T_2_, GG_W_H_2_, …, UG_W_H_3_. Sensors designated as UGwH were installed under the permeable brick pavement for measuring soil humidity. The soil water content under the pavement was high (Table 9). The underground temperature was also higher than that above ground, possibly because permeable bricks can absorb heat, resulting in the increase of soil temperature (Figure 6). Regarding temperature, from approximately 7:00 a.m., because of sunshine, the temperature began to increase. At 2:00–3:00 p.m., because of surface heat radiation, the temperature reached its daily peak. By approximately 7:00 p.m., the temperature had generally decreased. As for humidity, relative humidity was measured. Moreover, because the measurement subject was water vapor in the **air** and rising temperature increases the vapor content in the air, an approximately 30% decrease in relative humidity was observed. Permeable brick pavements were studied at two locations in this study. The permeable brick pavement in front of the Integrated Technology Complex was analyzed from 17 May to 23 May, during which sunny weather and strong sunshine prevailed. If measurement days were rainy with low sunshine intensity, the temperature curve would not have presented substantial changes, and similarities could not be utilized to confirm the similarity of the two curves. (When the two curves are horizontal lines, discussing their similarities is meaningless.) Relative humidity remained mostly at 99%. As displayed in Figure 6c, in that 1-week interval, the trends of the GGwT_2_, UGwT_2_, and the CWB Taipei Station curves were similar. Table 10 lists the pairwise comparison of the similarities of the curves. The other site was the permeable brick pavement near the Zhongxiao Gate. The temperature and humidity trend from 16 May to 22 May are presented in Figure 6d. The peak of the curve occurred at approximately 2:00 p.m.

**Figure 7:**
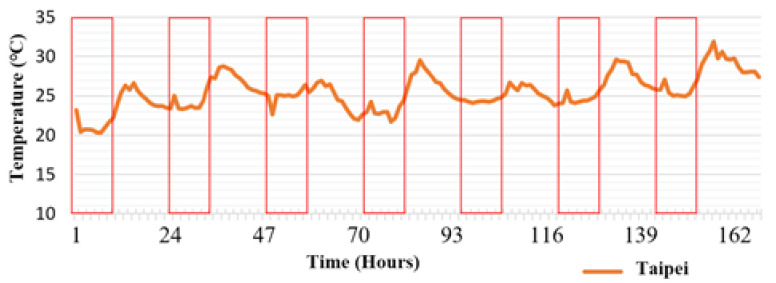
Time period used for calculating mean temperature differences.

**Figure 8:**
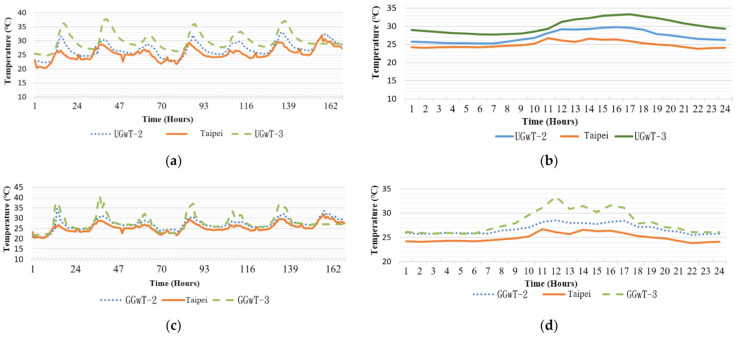
Temperature of Type A and Type B permeable brick pavement (**a**) 10 cm underground (**b**) partial observation on 21 May at 10 cm underground (**c**) above ground (**d**) partial observation on 21 May above ground.

Different regions were compared for the same time period. The mean temperature differences during the day were also calculated. Day time was set at 7:00 a.m. to 5:00 p.m., a total of 10 h. The calculation region is marked using a red square in Figure 7. Temperature curves in Figure 8a,b reveal that the temperature of Type B permeable brick pavement was higher that than of Type A. The mean difference was approximately 3.29 °C. Figure 8c,d present the temperature of the two regions above ground, where the temperature of Type B was also higher than that of Type A, by an average of 1.75 °C. The environment of the two regions was unshaded permeable pavement. The temperature above and below the ground covered by the Type A permeable brick pavement differed by an average of 0.8 °C. For Type B, the difference was an average of 2.34 °C. Clearly, Type B had a superior temperature reduction effect than Type A. Thus, permeable brick pavement was positively correlated with temperature reduction. 

### 3.3. Analysis of Data from Greening Rooftop

**Figure 9:**
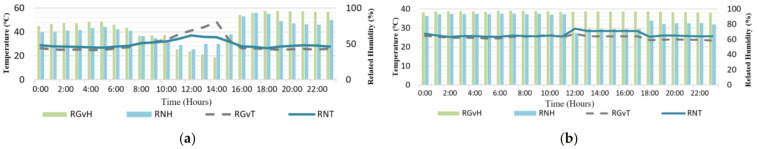

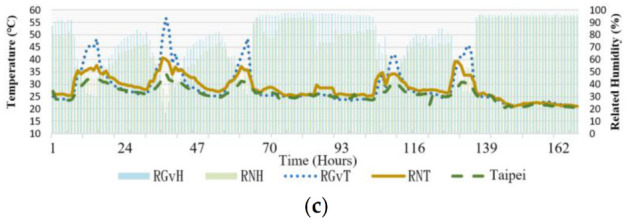
Temperature and humidity on the greening rooftop (**a**) on 12 May, (**b**) on 13 May, and (**c**) the temperature reduction effectiveness.

Figure 9a,b present data from two consecutive days: 12 May and 13 May. From 10:00 a.m. to 2:00 p.m., the values from RGvT, a region with plants, were higher than those of RNT. According to observation data from the CWB, at approximately 5:00 p.m. on 12 May, it rained. At that time, the temperature dropped to approximately 20 °C, and the humidity increased to 90%. The next day, 13 May, it also rained at times (e.g., at 1:00 a.m. and 6:00 a.m.); thus, the temperature and humidity exhibited a horizontal trend. At 12:00 p.m., the sunshine intensity increased, resulting in a decrease in the amount of water vapor in the air. Regarding the data in the region with plants, because the water vapor above the soil dissipated slower and because it did not rain afterward, the temperature exhibited a slight difference. Figure 9c presents the temperature and humidity data for the greening rooftop. The analyzed period was 7 days, from 10 May to 16 May. Curves depicting data from RGvT and RNT were compared. From 10:00 a.m. to 2:00 p.m., values from RGvT were higher than those of RNT. This phenomenon, observed on various days, may be attributed to the following factors: (1) the location of the sensor was exposed to the sun, resulting in a surge of the temperature indicated by the thermometer, or (2) the location was above the greening region instead of parallel to the plants, resulting in a substantial difference between the two. Table 11 presents the similarities in Region E. 

## 4. Discussion

### 4.1. Comparison of Regions at the Same Elevation

**Figure 10:**
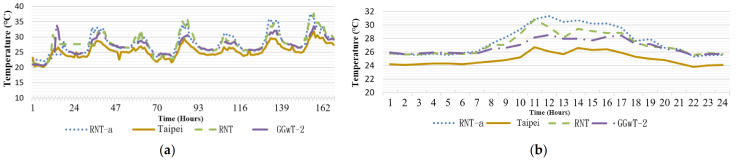
(**a**) Rooftop temperature without greening (**b**) partial observation on 21 May.

**Figure 11:**
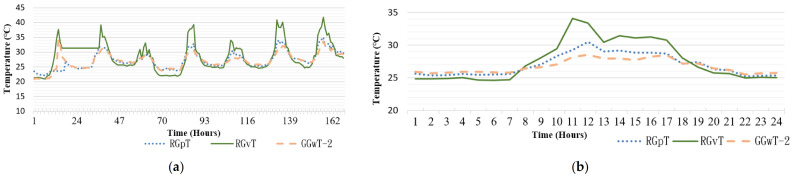
(**a**) Rooftop temperature with greening (**b**) partial observation on 21 May.

Regions B and E were approximately at the same elevation. The RNT (temperature on the rooftop region without greening) of the two regions was not affected by shade. Figure 10a reveals that the two regions had a temperature difference of 0.81 °C. According to Figure 10b, which depicts a partial observation duration, the temperature difference was more pronounced from 08:00 a.m. to 16:00 p.m., but the values were still similar. Because the environments of both regions were similar, the data from RNTa and RNT were similar. The temperatures recorded by the two sensors were 5 °C lower than that of the CWB (whose sensor was positioned at an elevation of 5 m and shaded). In Figure 11b, the temperatures of Regions B and C were similar, with a difference of approximately 1–2 °C. The temperature at locations with higher elevation was lower. 

### 4.2. Comparison of Various Greening Methods

In Figure 11, the values from RGvT considerably exceed those from RGpT and GGwT, and its temperature also exceeds that recorded in the nonshaded area. That is, RGvT exhibited a lower temperature reduction trend than RGpT and GGwT. Its temperature reduction effectiveness was unrelated to the greening method. Comparison of the curves in Figure 11a indicated that RGpT and RNTa exhibited a temperature difference of 1.51 °C, revealing that shade from solar panels can reduce temperature. GGwT also achieved a temperature reduction of 0.81 °C. From 6:00 p.m. to 7:00 a.m. the next day, the temperatures of all curves were identical. Therefore, they were not included in the calculation for mean temperature reduction effectiveness.

## 5. Conclusions

This study used a self-constructed, interdisciplinary integrated sensing system to monitor and quantify the effectiveness of the green engineering projects on campus. The sensing points were established at three green engineering projects on the West Campus of National Taipei University of Technology (the latitude and longitude are 121.5332~121.5360 and 25.0422~25.0431, separately) to examine the correlation between green engineering and reducing the temperature in the environment. The discussed green engineering projects involves solar **panel** shade, a permeable brick pavement, and a greening rooftop. The green smart campus was analyzed temporally and spatially. The observation periods were individual days and full weeks. The compared spatial aspects were various forms of permeable bricks, elevation, and green engineering projects. The results are as follows:

1. The monitoring and testing system design and establishment were aimed at universality. The components of all sensing dimensions, output signal types of the sensing elements, and sensing element calibration procedures were considered. A wireless transmission protocol featuring low power consumption and long-distance transmission was selected. Unlike previous related studies that selected a single research site and designed a sensing system specific for that site, the hardware and software employed in this research may be modified according to the precision and measurement requirements of various research objectives and different sites. Therefore, it can be applied in diverse fields. The transmission protocol used in this study was LoRa communication technology. It effectively overcame the limitation of wireless data transmission distance in past sensing systems, improving the transmission distance of 100–200 m in previous studies to a minimum of 1 km and up to 8 km. This technology also resolved the problem of wasting bandwidth.
2. A specific time period on one day was used to observe the temperature change in each green engineering project. At 7:00 a.m. that day, the temperature started to increase and the temperature recorded by the sensors began to differ. Regarding the changes in temperature over the course of that day, the highest temperature was recorded between 2:00 p.m. and 3:00 p.m. The soil moisture under the permeable brick pavement was high. In the afternoon, the temperature of the soil was also higher than that above ground. The temperature measured in the shade of the solar panels was lower than that in the unshaded area, indicating that the solar panels could mitigate the temperature increase on a building rooftop’s concrete surface. As for the region on the greening rooftop, the humidity sensor was placed above the soil, where water vapor effused slowly. The temperatures in each region indicated that the extent of temperature increase at the permeable brick pavement was smaller than that in other regions.
3. The temperature difference between areas with and without Type A permeable brick was 0.8 °C, and that of area with and without Type B permeable brick was 2.34 °C. Type B exhibited a better temperature reduction effect than Type A did. Permeable brick pavement was positively correlated with temperature reduction. At the same elevation, in areas with and without greening, the temperature difference was approximately 0.81 °C. The temperature at a lower elevation was 1–2 °C higher than that at a higher elevation.
4. The greening methods were compared. Shade provided by solar panels had a superior temperature reduction effect than did permeable brick pavement. Green engineering method was negatively correlated to temperature reduction. On the ground, permeable pavement and temperature reduction were positively correlated. The temperature reduction effectiveness of permeable pavement was poorer than that of shade provided by solar panels. The temperature difference between a high-rise building and the ground was approximately 1–2 °C. At the same elevation, the temperature difference was approximately 0.8 °C.

## Figures and Tables

**Table 1:** SHT10 temperature and humidity sensing element—humidity parameters.

Category		Typ	Unit
Humidity sensor			
Resolution		0.05	% RH
		12	bit
accuracy		±4.5	% RH
Hysteresis		±1	% RH
Non-linearity	Original value	±3	% RH
	Linearization value	<<1	% RH
Response time		8τ	sec
Operating range		0~100	% RH
Long term drift		<0.5	% RH/yr
Temperature sensor			
Resolution		0.01	°C
		14	bit
accuracy		±0.5	°C
Repeatability		±0.1	°C
Response time		5τ~30τ	sec
Operating range		−40~123.8	°C
Long term drift		<0.04	°C/yr
Electrical spec			
Supply voltage range		2.4~5.5	V
Power consumption	Sleep state	2	µW
	Measurement state	3	mW
Supply current	High signal transmission state	220	µA
	Low signal transmission state	55	µA
	Sleep state	0.6	µA

**Table 2:** Temperature interval data of Sensor 1.

Region	Sense 1 Temperature (°C)	Testing Temperature (°C)	Difference	①	②	③
1	30.34	29.12	1.22	233.35	234.26	232.43
2	35.85	33.87	1.98	102.78	95.89	110.16
3	40.36	38.67	1.69	30.11	27.95	32.44
4	45.84	44.3	1.54	(0.01)	0.04	0.00
5	50.64	49.6	1.04	26.14	24.94	27.40
6	55.70	54.8	0.90	104.92	101.11	108.87
7	60.79	60.2	0.59	239.75	229.26	250.72
mean	45.64	44.37		737.04	713.46	762.03
$\gamma_{R}$			0.999583329		26.71	27.60

**Table 3:** Module specification table.

Parameter		Value	Unit
Band		410~441	Hz
Maximum transmission distance		8000	m
Transmission speed		0.3, 1.2, 2.4, 4.8, 9.6, 19.2	kbps
Transmission power		30, 27, 24, 21	dBm
Baud rate (for selection)		1200~115,200	-
Packet length		512	byte
Working temperature		−40~+85	°C
Working humidity		10~90	%RH
Antenna format		SMA-K	-
Supply voltage	Rated voltage	3.3~5.5	V
	High potential	5.2	V
	Low potential	0	V
Supply current	Transmission current	670@30 dBm	mA
	Receiving current	14.5	mA
	Sleep current	3	μA

**Table 4:** Power consumption in power-saving mode.

Supply current	Transmission current	670@30 dBm	mA
	Receiving current	14.5	mA
	Sleep current	3	μA

**Table 5:** Parameters meeting the requirements of the research site.

Frequency	440 MHz	Baud rate	9600
Address	0 × 0000	Serial format	8N1
Transmission speed	2.4 kbps	Transmission power	1 W
Channel	0 × 17	-	-

**Table 6:** Sensor serial number.

Region	Sensor Code	Description
A		Data collection hot spot devices
B	RG_p_T	Temperature in the shade on the rooftop
	RG_p_H	Humidity in the shade on the rooftop
	RNT	Temperature outside the shade on the rooftop
	RNH	Humidity outside the shade on the rooftop
C	GG_W_T_2_	Temperature 10 cm underground
	GG_W_H_2_	Humidity 10 cm underground
	UG_W_T_2_	Temperature above ground
	UG_W_H_2_	Humidity above ground
D	GG_W_T_3_	Temperature 10 cm underground
	GG_W_H_3_	Humidity 10 cm underground
	UG_W_T_3_	Temperature above ground
	UG_W_H_3_	Humidity above ground
E	RG_V_T	Temperature in the greening area
	RG_V_H	Humidity in the greening area
	RNT	Temperature outside the greening area
	RNH	Humidity outside the greening area
G_p_: Green of PV part G_W:_ Green of Water part Gv: Green of Vegetable green part		

**Table 7:** Region B: Data collected on the reduction in temperature under solar panel shade.

5/10	40002	40003	40004	40005	40018	40019
	RGpT	RGpH	RNT-a	RNH-a	RNT-b	RNH-b
	LAST	LAST	LAST	LAST	LAST	LAST
0:00	26.07	80.24	26.18	75.91	26.45	79.42
1:00	25.37	82.6	25.54	77.9	25.8	81.81
2:00	25.48	82.82	25.56	78.01	25.87	81.97
3:00	25.56	81.35	25.73	76.56	25.94	80.45
4:00	25.31	81.49	25.44	76.69	25.61	80.59
5:00	25.24	81.33	25.33	76.58	25.56	80.48
6:00	25.42	80.79	25.99	74.33	26.24	77.95
7:00	25.99	78.54	28.09	66.94	28.36	70.17
8:00	29.18	69.57	31.58	57.87	32.02	59.61
9:00	31.36	63.54	34	52.5	35.08	52.02
10:00	33.14	57.38	35.6	48.06	37.26	45.9
11:00	34.41	51.51	35.2	46.84	36.46	45.49
12:00	35.46	46.71	36.57	41.93	37.68	41.07
13:00	36.2	46.69	37.55	41.48	38.09	41.54
14:00	36.34	50.08	37.66	44.71	38.74	43.52
15:00	35.78	51.96	37.66	44.95	38.57	44.05
16:00	34.4	55.9	36.46	47.93	36.93	48.33
17:00	33.92	53.31	35.42	46.8	36.09	46.61
18:00	32.02	61.77	32.54	57.63	32.72	59.3
19:00	30.67	63.7	30.93	59.74	31.09	61.85
20:00	30.62	65.47	30.77	61.41	31.01	63.82
21:00	29.74	68.05	29.94	63.76	30.1	66.52
22:00	29.42	69.46	29.64	64.98	29.8	67.89
23:00	29.45	69.7	29.58	65.33	29.8	68.24

**Table 8:** Region B: Similarities of solar panel shading.

Curve	Taipei × RGpT	Taipei × RNTa	RGpT × RNTa
Similarity	99.9133%	99.8382%	99.9636%

**Table 9:** Regions C and D: Temperature changes in the permeable brick pavement.

	40006	40007	40008	40009	40014	40015	40016	40017
	GGwT-3	GGwH-3	UGwT-3	UGwH-3	GGwT-2	GGwH-2	UGwT-2	UGwH-2
	LAST	LAST	LAST	LAST	LAST	LAST	LAST	LAST
0:00	26.4	81.77	27.56	96.09	26.04	79.11	26.27	93.89
1:00	25.67	84.14	27.2	96.08	25.38	81.65	25.97	94.44
2:00	25.38	85.72	26.98	96.08	25.29	81.57	25.72	94.67
3:00	25.58	84.66	26.82	96.08	25.51	80.87	25.52	94.85
4:00	25.42	83.84	26.78	96.11	25.35	79.98	25.53	94.97
5:00	25.11	85.09	26.63	96.1	24.92	82.49	25.27	95.11
6:00	25.19	85.06	26.44	96.08	24.92	82.77	25.2	95.14
7:00	26.13	82.32	26.46	96.11	25.94	78.48	25.28	95.16
8:00	28.5	76.27	26.64	96.1	28.36	71.8	25.7	94.85
9:00	30.37	72	27.04	96.15	30.1	66.21	27.03	94.02
10:00	32.61	64.53	27.72	96.19	31.06	64.37	27.84	94.17
11:00	37.27	51.08	31.58	96.38	32.62	55.96	28.4	94.33
12:00	38.16	46.92	35.4	95.76	34.05	50.43	29.34	94.11
13:00	36.93	48.97	37.64	95.29	36.24	45.93	32.12	92.98
14:00	38.61	47.85	38.37	95.38	38.76	42.79	33.67	92.24
15:00	37.7	50.97	40.2	95.26	33.82	56.93	34.18	93.97
16:00	35.24	55.75	40.47	95.24	33.36	56.32	32.75	94.8
17:00	34.83	53.96	38.91	95.51	33.04	55.09	32.12	95.25
18:00	32.06	64.5	36.14	95.87	31.6	63.9	31.07	95.62
19:00	30.82	64.03	34.01	96.1	30.64	62.29	30.09	96.03
20:00	30.32	68.15	32.71	96.25	30.32	65.53	29.7	96.07
21:00	29.71	69.95	31.7	96.27	29.7	67.31	29.05	96.17
22:00	29.4	71.54	31.1	96.32	29.26	68.7	28.55	96.22
23:00	29.06	73.72	30.6	96.36	28.68	70.5	28.4	96.23

**Table 10:** Regions C and D: Similarities between permeable brick pavements.

Curve	Taipei × GGwT2	Taipei × UGwT2	UGwT2 × GGwT2
Similarity	99.9282%	99.8863%	99.9172%
**Curve**	**Taipei × GGwT3**	**Taipei × UGwT3**	**GGwT3 × UGwT3**
Similarity	99.6649%	99.7355%	99.5165%

**Table 11:** Region E: Sensor similarities on the greening rooftop.

Curve	Taipei × RNT	Taipei × RGvT	RNT × RGvT
Similarity	99.7797%	98.8781%	99.2350%

## Data Availability

The data presented in this study are available in this study.
